# Serotonin-prefrontal cortical circuitry in anxiety and depression phenotypes: pivotal role of pre- and post-synaptic 5-HT1A receptor expression

**DOI:** 10.3389/fnbeh.2014.00199

**Published:** 2014-06-06

**Authors:** Paul R. Albert, Faranak Vahid-Ansari, Christine Luckhart

**Affiliations:** ^1^Neuroscience, Ottawa Hospital Research Institute, University of OttawaOttawa, ON, Canada; ^2^Department of Cellular and Molecular Medicine, University of OttawaOttawa ON, Canada

**Keywords:** anxiety, depression, interneurons, pyramidal neurons, prefrontal cortex, raphe nuclei, serotonin receptors, transcription factors

## Abstract

Decreased serotonergic activity has been implicated in anxiety and major depression, and antidepressants directly or indirectly increase the long-term activity of the serotonin system. A key component of serotonin circuitry is the 5-HT1A autoreceptor, which functions as the major somatodendritic autoreceptor to negatively regulate the “gain” of the serotonin system. In addition, 5-HT1A heteroreceptors are abundantly expressed post-synaptically in the prefrontal cortex (PFC), amygdala, and hippocampus to mediate serotonin actions on fear, anxiety, stress, and cognition. Importantly, in the PFC 5-HT1A heteroreceptors are expressed on at least two antagonist neuronal populations: excitatory pyramidal neurons and inhibitory interneurons. Rodent models implicate the 5-HT1A receptor in anxiety- and depression-like phenotypes with distinct roles for pre- and post-synaptic 5-HT1A receptors. In this review, we present a model of serotonin-PFC circuitry that integrates evidence from mouse genetic models of anxiety and depression involving knockout, suppression, over-expression, or mutation of genes of the serotonin system including 5-HT1A receptors. The model postulates that behavioral phenotype shifts as serotonin activity increases from none (depressed/aggressive not anxious) to low (anxious/depressed) to high (anxious, not depressed). We identify a set of conserved transcription factors including Deaf1, Freud-1/CC2D1A, Freud-2/CC2D1B and glucocorticoid receptors that may confer deleterious regional changes in 5-HT1A receptors in depression, and how future treatments could target these mechanisms. Further studies to specifically test the roles and regulation of pyramidal vs. interneuronal populations of 5-HT receptors are needed better understand the role of serotonin in anxiety and depression and to devise more effective targeted therapeutic approaches.

## Introduction

### Depression and anxiety: roles of serotonin and prefrontal cortex (PFC)

Depression and anxiety are complex and heterogeneous disorders with a global disease burden that is steadily increasing and has currently surpassed most other major diseases (Whiteford et al., 2013). Their onset occurs during childhood to adolescence persisting throughout lifetime. Depression and anxiety are often comorbid and display a relapsing and remitting course that becomes resistant to pharmacologic treatment (Gorwood, 2004; Trivedi et al., 2006; Krishnan and Nestler, 2008; Rush et al., 2009). Depression is complex in part because the diagnostic criteria do not distinguish between different behavioral facets of depression, hence very different subsets of depressed patients are “lumped” together (Krishnan and Nestler, 2008). Nevertheless, twin studies demonstrate a clear genetic component, and a large number of studies have implicated gene x environment interactions as a key determinant of depression primates (Caspi et al., 2010). Prominently adverse, stressful environment in early post-natal or adolescent life increases the likelihood of depression and the strength of association of genetic variant with depression.

Several lines of evidence implicate decreased serotonergic activity in anxiety and major depression, but its importance in the etiology and severity of these disorders remains unclear (Millan, 2004; Wong et al., 2005; Jans et al., 2007; aan het Rot et al., 2009; Booij et al., 2010). Acute tryptophan depletion studies support a role for decreased serotonin in depression, or at least in relapse of recovered depressed patients (Barnes and Sharp, 1999; Young and Leyton, 2002; Jans et al., 2007; Lanfumey et al., 2008; aan het Rot et al., 2009). Imaging and post-mortem studies indicate altered activity of discrete regions within the prefrontal cortex (PFC) and their atrophy are associated with depression (Rajkowska et al., 2005; Ressler and Mayberg, 2007). Genetic polymorphisms in serotonin genes [e.g., 5-HT transporter (5-HTT), 5-HT1A receptor] have been associated with depression, but these associations are weak and not always reproducible, suggesting that serotonin may be a predisposing factor rather than a cause of depression or anxiety (Karg et al., 2011; Kishi et al., 2013). The mainstay of pharmacological treatment targets serotonin and/or other monoamine systems, but these approaches have very modest treatment effects and initial monotherapy with serotonin-specific reuptake inhibitor (SSRI) is ineffective to achieve sustained remission in a majority of patients (Trivedi et al., 2006; Rush et al., 2009). More recently the finding that ketamine can induce rapid antidepressant effect coincident with remodeling and spine development in the PFC suggest a key role for the PFC in depression and its treatment (Aan Het Rot et al., 2012; Duman and Li, 2012). Deep brain stimulation of discrete sites within the PFC has elicited remarkable improvement in depression symptoms in some depressed patients, supporting the key role for hypo/hyper-activity of different regions of the PFC in depression (Ressler and Mayberg, 2007; Holtzheimer and Mayberg, 2011). Interestingly, the beneficial effects of deep brain stimulation in animal models of depression depend on 5-HT, indicating a primary role of the 5-HT-PFC circuitry in effective treatment of depression (Hamani and Nobrega, 2012). In this review, we focus on the role of pre- and post-synaptic 5-HT1A receptors in regulating the activity of this circuitry, and on the behavioral outcomes of modifying this circuitry.

### The serotonin system: roles of pre- and post-synaptic receptors

The brain serotonin system consists of a small group of neurons located in the raphe nuclei of the midbrain that are unique since they express the rate-limiting enzyme for serotonin synthesis, tryptophan hydroxylase-2 (TPH2), the brain specific isoform Walther et al., 2003; Lenicov et al., 2007). The differentiation of neuronal progenitors to express serotonergic markers like TPH2 is driven by the transcription factor Pet-1, which directly activates the TPH2 gene (Hendricks et al., 2003). 5-HT neurons of the rostral raphe nuclei, including the dorsal and median raphe nuclei, project widely throughout the brain to innervate key brain regions involved in anxiety and depression. Activation of serotonin neurons induces release of serotonin at target neurons and within the raphe via collateral branches (Kocsis et al., 2006; Bang et al., 2012), which is rapidly removed by the 5-HTT, the target of SSRI antidepressants. Serotonin released in the raphe activates the 5-HT1A autoreceptor, which negatively regulates the firing of serotonin system. Release of serotonin at target neurons activates 5-HT heteroreceptors including the 5-HT1A heteroreceptor which is abundantly expressed in the hippocampus, septum, amygdala, and PFC (Albert et al., 1990) where it mediates serotonin actions on fear, anxiety, stress, and cognitive function (Barnes and Sharp, 1999; Gross and Hen, 2004; Savitz et al., 2009; Meltzer et al., 2012; Donaldson et al., 2013; Garcia-Garcia et al., 2013). Since the 5-HT1A receptor is coupled to inhibitory Gi/Go proteins (Barnes and Sharp, 1999; Albert and Tiberi, 2001), it inhibits the firing and activity of target neurons (Celada et al., 2004; Puig and Gulledge, 2011; Llado-Pelfort et al., 2012). The other major 5-HT receptor (particularly in cortex) is the Gq-coupled 5-HT2A receptor, which mediates excitatory actions of serotonin on target neurons (Celada et al., 2004; Puig and Gulledge, 2011; Llado-Pelfort et al., 2012). Thus in terms of neural circuitry, the 5-HT1A receptor is inhibitory, while the 5-HT2A receptor is stimulatory. However, these receptors can also couple to protein kinase pathways in a cell-specific manner that may stimulate gene transcription and indirectly enhance neuronal function in the long term (Kushwaha and Albert, 2005; Mogha et al., 2012). For the purpose of clarity, we focus on the 5-HT1A as the major inhibitory receptor and 5-HT2A as the major stimulatory receptor in PFC, although there are complementary roles for other stimulatory 5-HT receptors in PFC, such as 5-HT3, 5-HT4, and 5-HT7 receptors (Beique et al., 2004).

## Targeted genetic modification of the serotonin system and behavior

### Transgenic and knockout approaches

Several mouse models have targeted the 5-HT1A receptor gene or genes involved in determining 5-HT levels or 5-HT neurotransmission in the brain (Table 1) (Jacobsen et al., 2012a; Lesch et al., 2012; Donaldson et al., 2013). More recently these studies have included conditional and inducible knockout or suppression approaches summarized in Box 1. Gene knockout approaches result in irreversible loss of gene function, while inducible suppression or expression approaches allow for reversible expression. Inducible expression of CRE (for inducible knockout) has mainly been done use the CRE-ERT2 construct, which is activated by tamoxifen treatment. Inducible suppression has been done using a doxycycline-suppressible repressor construct (Tanaka et al., 2010). By the use of tissue-specific promoters, these approaches can target specifically serotonin neurons (using Pet-1 or TPH2 promoters) or forebrain regions (usually CAMKIIα promoter). The Pet-1/TPH2 promoters have been used to drive expression of CRE recombinase in all midbrain serotonin neurons from the initiation of their differentiation, with TPH2 driving somewhat stronger expression. On the other hand, the CAMKII promoter drives expression starting at the early post-natal period and is widely expressed in glutamatergic pyramidal neurons of the forebrain but excluded from interneurons (Xu et al., 2000; Chen et al., 2010). Together, these approaches provide both region- and cell type-specific gene targeting that can be induced or reversed at different developmental times.

**Table 1:** **5-HT genetic models summary**.

**Genetic model**	**Target gene expression**			**Adult behavior**	**References**
	**Embryo (%)**	**Early PN (%)**	**Adult (%)**		
**5-HT1A**					
5-HT1A^−/−^	−	–	−	Anxiety, antidepressed	Heisler et al., 1998; Parks et al., 1998; Ramboz et al., 1998
5-HT1A auto-iS (Pet1-tTX)	+	+	−30	Antidepressed, SSRI+	Richardson-Jones et al., 2010
5-HT1A auto-iS (Pet1-tTX)	+	−40	+	Anxiety, social−	Donaldson et al., 2014
5-HT1A auto-iS (Pet1-tTX)	−80	−80	−80	Anxiety	Richardson-Jones et al., 2011
5-HT1A hetero-iS (CamKII-tTX)	−95	−95	−95	Depression	Richardson-Jones et al., 2011
5-HT1A auto-siRNA	+	+	−80	Anti-depressed, SSRI+	Bortolozzi et al., 2012; Ferres-Coy et al., 2013
5-HT1A hetero-rescue (CaMKII-tTA iTg)	−	+	−	Anti-anxiety	Gross et al., 2002
5-HT1A-heteroOE (5-HT1A-tg)	+	++	+	Anti-anxiety, Anti-depressed	Kusserow et al., 2004
5-HT1A-autoOE (Tph2-tg)	+300	+300	+300	Aggression, anxiety	Audero et al., 2013; Piszczek et al., 2013
5-HT1A-auto rescue (Tph2-tg)	+300	+300	+300	Anxiety, anti-depressed	Audero et al., 2013; Piszczek et al., 2013
**5-HT**					
TPH2^−/−^	−	–	−	Anti-Anxiety, depression, aggression	Mosienko et al., 2012
TPH2-R439H KI	−80	−80	−80	Anxiety, depression	Jacobsen et al., 2012b; Sachs et al., 2013
PET1^−/−^	−80	−80	−80	Aggression, anxiety	Hendricks et al., 2003
Pet1-adult-iKO	+	+	−80	Anxiety	Liu et al., 2010
En1/Pet1-TetTX IT	−^*^	−^*^	−^*^	Anti-Anxiety, cognitive+	Kim et al., 2009
**5-HTT**					
5-HTT-/-	−	–	−	Anxiety, depression anti-aggression	Holmes et al., 2002, 2003; Lira et al., 2003; Kalueff et al., 2006
5-HTT-FLX	+	−	+	Anxiety	Ansorge et al., 2004
5-HTT-G56A KI	++^*^	++^*^	++^*^	Social−, SSRI+	Veenstra-Vanderweele et al., 2012
5-HTT-tg	+100	+100	+100	Anti-anxiety	Jennings et al., 2006

Genetic models are as defined in Box 1 with promoter used indicated in parentheses; rescue is expression in knockout background, over-expression (OE) is on wild-type background; auto, autoreceptor-specific; hetero, heteroreceptor-specific. The effect of the indicated genetic model on target gene expression (−, none; +, normal; ++, increased; +X, increased by X%) at indicated developmental time [embryo, early post-natal (PN) or adult] and adult behavior phenotype or response to SSRI are indicated.

* loss of 5-HT release; enhanced activity 5-HTT mutant.

Box 1 **List of genetic manipulations.**

**Genetic models presented in Table 1 are summarized below**

**Transgenic (Tg):** Promoter-gene; Promoter dependent expression of gene; non-reversible

**Knockout (KO, ^−/−^):** Gene disrupted; ubiquitous; lifetime; non-reversible

**Conditional KO (cKO):** flx-gene-flx X Promoter-CRE: Gene disrupted in CRE-expressing cells only; promoter-dependent onset; non-reversible

**Conditional induction:** flx-STOP-flx-gene X Promoter-CRE: Gene induced in CRE-expressing cells; promoter-dependent onset; non-reversible

**Inducible Knockout (iKO):** flx-gene-flx X Promoter-CRE-ERT2: Gene disrupted upon treatment (tamoxifen) in CRE-expressing cells only; age-specific; non-reversible

**Inducible Transgenic (iTg):** TetO-KI-gene X Promoter-tTA; Promoter- and doxycycline regulated expression of gene; tissue-specific; promoter-dependent onset; reversible with doxycycline

**Inducible Suppression (iS):** TetO-KI-geneI X Promoter-tetR-KRAB; Promoter- and doxycycline regulated suppression of gene; tissue- specific; promoter-dependent onset; reversible with doxycycline treatment

**Knockin (KI):** Recombination of mutant gene for w.t.; normal gene expression pattern retained; non-reversible

**Intersectional Transgenic (IT):** flx-STOP-flx-flp-STOP2-flp-gene x promoter1-CRE x promoter2-FLP: gene expression dependent on inter section of promoters 1 and 2; promoter-dependent onset; non-reversible

**siRNA suppression:** siRNA depletes target gene; can be targeted to 5-HT neurons by fusion to SSRI; reversible within few days.

### Rodent behavioral readouts of anxiety, depression and aggression

These rodent genetic models have been examined for 5-HT neurotransmission and for anxiety, depression, and aggression-related behaviors using a standard set of assays (Table 1). Anxiety behavior has been typically assayed using elevated plus maze (Lister, 1987), light-dark box, open field tests (Bouwknecht et al., 2004; Crawley, 2008), which measure acute anxiety-like behavior. In some cases novelty-suppressed feeding assay, which has greater face validity since it responds to chronic and not acute SSRI treatment (Santarelli et al., 2003). Depression-like behavior has mainly been studied using acute tests like tail suspension (Liu and Gershenfeld, 2003) or forced swim tests (Porsolt et al., 1977; Castagne et al., 2009). These tests measure acute depression or behavioral despair in an inescapable stress and are used to identify antidepressant compounds. In a few cases, the sucrose preference test has been done to assess anhedonia (Krishnan et al., 2008). Aggression has been evaluated using the resident-intruder test, and social interaction with animal vs. object or novel animal was quantified. These tests assess limited phenotypes compared to the more complex phenotypes of anxiety disorders or major depression in humans. Thus the circuitry models presented below are in the context of these assays of anxiety- or depression-like behavior that may correspond to discrete endophenotypes of anxiety disorder or depression in humans.

## Serotonin-PFC circuitry in anxiety and depression rodent models

### Serotonin-PFC circuitry model in the anxiety phenotype

**Figure 1:**
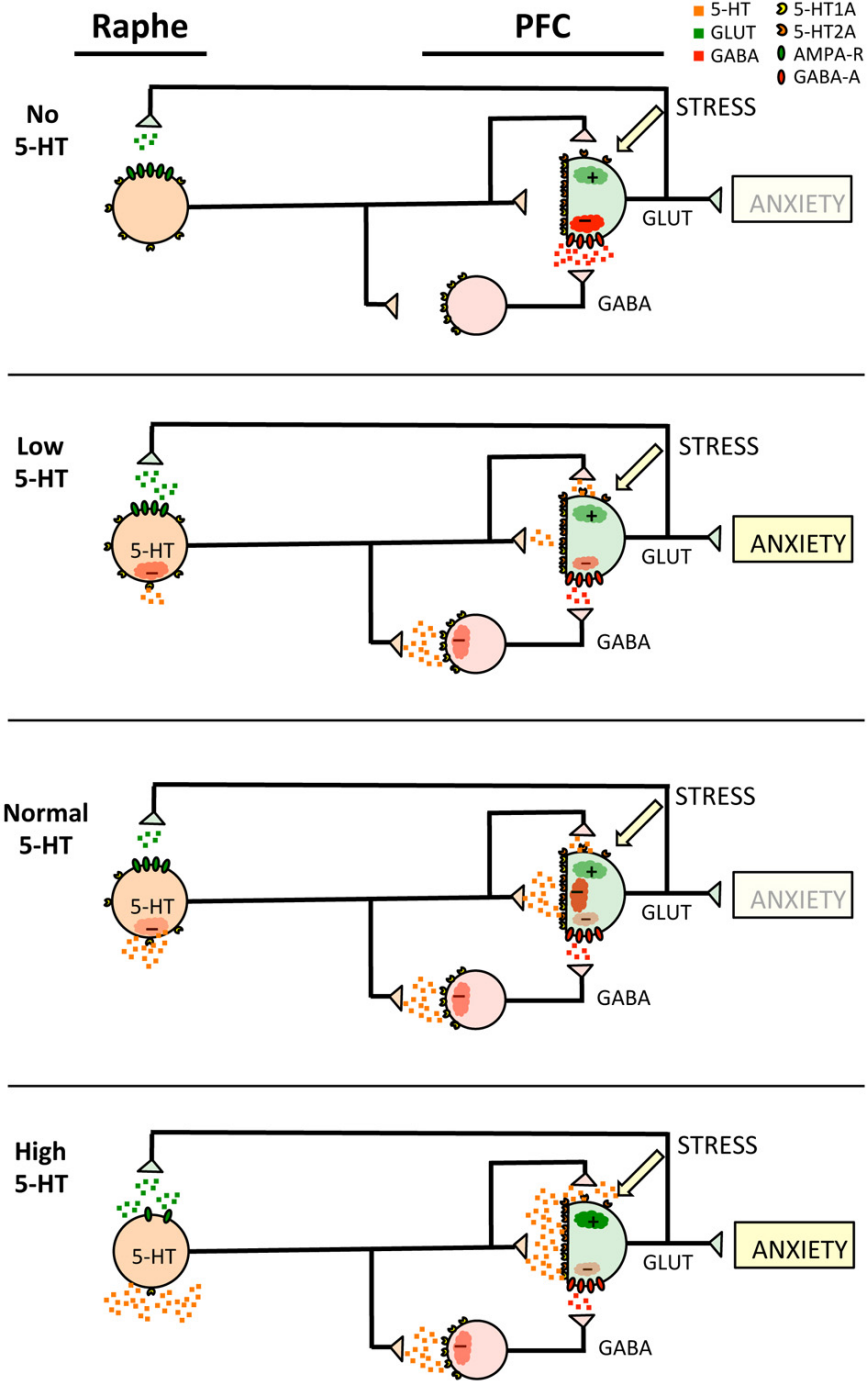
**Early post-natal 5-HT-PFC circuitry in anxiety models**. Shown are the components of the Raphe 5-HT-PFC circuit in animals during the early post-natal period with no, low, normal or high levels of 5-HT neurotransmission as indicated. Evidence from genetic mouse models (Table 1) supports the importance of alterations in early post-natal circuitry in generating the adult anxiety phenotype. The model shows 5-HT neurons (orange) projecting to prefrontal cortex GABAergic interneurons (red) and glutamatergic pyramidal neurons (green) with transmitter release illustrated as small squares of the same colors. Although 5-HT neurons are shown projecting separately to glutamatergic or GABAergic PFC neurons to illustrate the different activities of these pathways, the same 5-HT neuron may innervate both cells with different efficiency synapses or varicosities (Bang et al., 2012). The presence of 5-HT1A (yellow), 5-HT2A (orange) and GABA-A receptors (red) is shown, as well as the response in the target neurons (clouds), stimulatory (+) or inhibitory (−). The effect of chronic stress to stimulate pyramidal output is also indicated. It is postulated that 5-HT1A-mediated inhibition in the early post-natal period and perhaps adult is greater in interneurons than pyramidal neurons and that increased pyramidal neuronal activity triggers the anxiety phenotype, especially during the early post-natal period.

Comparing different genetic models, the effects of modifications of the serotonin system on anxiety behavior may often appear paradoxical or in conflict (Table 1), but can be in part accounted for by the model of 5-HT-PFC circuitry presented in Figure 1. This model takes into account that there are two major sub-populations of heteroreceptors in the PFC: 5-HT1A receptors on pyramidal glutamatergic neurons that inhibit their activity; and 5-HT1A receptors on interneurons that reduce inhibition to enhance pyramidal neuron activity (Amargos-Bosch et al., 2004; Santana et al., 2004). Immunostaining has identified 5-HT1A receptors on human pyramidal axon initial segment and parvalbumin positive chandelier cells in layer II/III (DeFelipe et al., 2001); pyramidal neurons in layer 2 tree shrew (Palchaudhuri and Flugge, 2005). 5-HT1A receptor RNA is present in 80% of glutamatergic neurons in external layers II and upper III, and in around 50% in layer VI (de Almeida and Mengod, 2008). Electrophysiological studies show presence of 5-HT1A receptors in pyramidal and interneurons, the latter mediating inhibition of pyramidal activity (Amargos-Bosch et al., 2004; Santana et al., 2004). In cortical neurons, 5-HT1A receptor activation inhibits NMDA currents (Yuen et al., 2008), and reduces CAMKII-induced AMPA phosphorylation (Cai et al., 2002), resulting in an inhibition of neuronal activity.

Critical aspects of the serotonin circuit model in anxiety (Figure 1) are:

- The hypothesized greater sensitivity of 5-HT1A receptors on interneurons compared to relatively lower sensitivity of 5-HT1A receptors on glutamatergic pyramidal neurons that mediate the anxiety phenotype;
- the greater activity of 5-HT2A vs. 5-HT1A receptors located on pyramidal neurons during the early post-natal period compared to adult (Beique et al., 2004) that once activated 5-HT2A receptors antagonize 5-HT1A signaling and stimulate glutamatergic output (Celada et al., 2004; Puig and Gulledge, 2011; Llado-Pelfort et al., 2012);
- increasing activity of this pyramidal projection increases anxiety.

The evidence for a role of 5-HT1A receptors on interneurons is mainly from systemic agonist (8-OHDPAT) administration studies in anaesthetized rats: DPAT reduces interneuron firing and increases pyramidal cell firing (blocked by GABAzine) (Llado-Pelfort et al., 2012). Pharmacological depletion of endogenous 5-HT in PFC to 5% of normal, reduced pyramidal firing from about 2 Hz to 1 Hz, suggesting that interneuronal 5-HT1A inhibition may play the predominate role in pyramidal activity under basal anaesthetized conditions. However, the role of pyramidal vs. interneuron 5-HT1A receptors in pyramidal firing activity needs to be tested in awake behaving conditions using specific blockade or knockout of these subpopulations of 5-HT1A receptors. There is also evidence for a developmental switch in 5-HT-induced responses in pyramidal neurons (Beique et al., 2004). Although 5-HT2A receptors have 10-fold lower affinity for 5-HT, in early post-natal development 5-HT excites PFC pyramidal cells via activation of highly expressed 5-HT2A (and 5-HT7) receptors, while 5-HT1A receptors are weakly coupled. With maturation, 5-HT1A receptor inhibition predominates over 5-HT2A function (which becomes desensitized) as rats mature from early post-natal period to adulthood.

In the anxiety model (Figure 1), under normal conditions both 5-HT1A heteroreceptors on pyramidal and interneurons are engaged, resulting in a balance between 5-HT1A-mediated inhibition and dys-inhibition of pyramidal anxiety output neurons. In mice with complete or nearly complete loss of 5-HT (Figure 1, No 5-HT; Table 1, TPH2 or TPH1/2 double knockout), 5-HT1A inhibition of both pyramidal and interneurons is predicted to be inactive and tonic interneuronal inhibition of pyramidal neurons to predominate, thereby reducing anxiety. Similarly, in mice expressing tetanus toxin in 5-HT neurons there is a similar outcome of reduced anxiety since release of 5-HT is prevented, thus mimicking a lack of 5-HT. In models where 5-HT is reduced but not entirely eliminated (Figure 1, Low 5-HT; Table 1, Pet-1^−/−^, TPH-R439H knockin) the model predicts that high affinity 5-HT1A receptors on interneurons are preferentially engaged compared to those on pyramidal cells, relieving GABA-mediated inhibition of pyramidal neurons in the anxiety circuit, resulting in over-activation by even mild stressors and an elevated anxiety phenotype (Holmes, 2008). Similarly, over-expression of 5-HT1A autoreceptors also reduces 5-HT activity leading to a hyperactive anxiety circuit by preferentially relieving inhibition of pyramidal neurons. On the other hand, global knockout of all 5-HT1A receptors would allow for hyper-activation of raphe neurons (due to absence of 5-HT1A autoreceptor) and activation of pyramidal 5-HT2A receptors that is not antagonized by pyramidal 5-HT1A receptors due to their homologous and heterologous (5-HT2A-mediated) desensitization, leading to increased anxiety. Pyramidal 5-HT2A receptors may also actively suppress interneuronal GABA-mediated inhibition via activation of protein kinase C (Bright and Smart, 2013). Consistent with a pro-anxiety role, mice lacking 5-HT2A receptors display reduced anxiety that is reversed by Emx1-driven cortical re-expression of 5-HT2A receptors (Weisstaub et al., 2006). In addition, 5-HT2A receptor levels are increased in early life stress and confer vulnerability to PFC hyperactivation and anxiety (Benekareddy et al., 2010). Consistent with this model, blockade of 5-HT2A receptors reduces anxiety induced by post-natal SSRI, while post-natal blockade of 5-HT1A receptors induces anxiety (Sarkar et al., 2013). Oppositely, early post-natal CaMKIIα-driven rescue of 5-HT1A heteroreceptors on pyramidal neurons in the 5-HT1A^−/−^ background rescues the anxiety phenotype (Gross et al., 2002). Early post-natal over-expression of the 5-HT1A receptor in wild-type background also reduced anxiety (Kusserow et al., 2004). On the other hand, CaMKIIα-driven promoter suppression of 5-HT1A receptors on pyramidal neurons (Richardson-Jones et al., 2011) may have had little effect on anxiety due to the maintained inhibition by GABAergic neurons. Thus, upon hyper-activation of the 5-HT system (High 5-HT, Figure 1), interneurons are predicted to be suppressed by interneuronal 5-HT1A activation while 5-HT2A receptors are predicted to respond to high 5-HT to activate pyramidal neurons and desensitize pyramidal 5-HT1A and GABA signaling via protein kinase C activation (Lembo and Albert, 1995; Wu et al., 2002), leading to a hyper-activated pyramidal activity and an anxiety phenotype.

The 5-HT1A receptor seems to play its major role in establishing the anxiety phenotype during the early post-natal period. For example, CaMKIIα-driven promoter rescue of 5-HT1A receptor expression in adult 5-HT1A^−/−^ mice failed to restore normal anxiety (Gross et al., 2002). Similarly, suppression of 5-HT1A autoreceptor expression during the early post-natal period but not adulthood elicited anxiety phenotype (Richardson-Jones et al., 2010; Donaldson et al., 2014). This critical period for the anxiety phenotype is associated with a developmental increase in the activity of 5-HT1A receptors on pyramidal neurons during this period (Beique et al., 2004). Suppressing pyramidal 5-HT1A signaling by hyper-stimulating 5-HT2A receptors or by partially reducing 5-HT activity leads to the hyper-activation of pyramidal neurons to drive the anxiety phenotype. Anxiety in the 5-HT1A knockout mouse appears to be resistant to the over-expression in adulthood of 5-HT1A receptors in pyramidal cells, suggesting that the predominance of 5-HT2A signaling prevents the inhibitory function of 5-HT1A receptors. Interestingly early life stress increases 5-HT1A RNA and 5-HT1A-induced potassium current in the PFC, which may be a protective adaptation that gradually diminishes with maturation to adulthood (Goodfellow et al., 2009); while 5-HT2A receptor signaling is also increased resulting in increased serotonin excitability of these neurons (Benekareddy et al., 2010).

### Serotonin-PFC circuitry model in the depression phenotype

**Figure 2:**
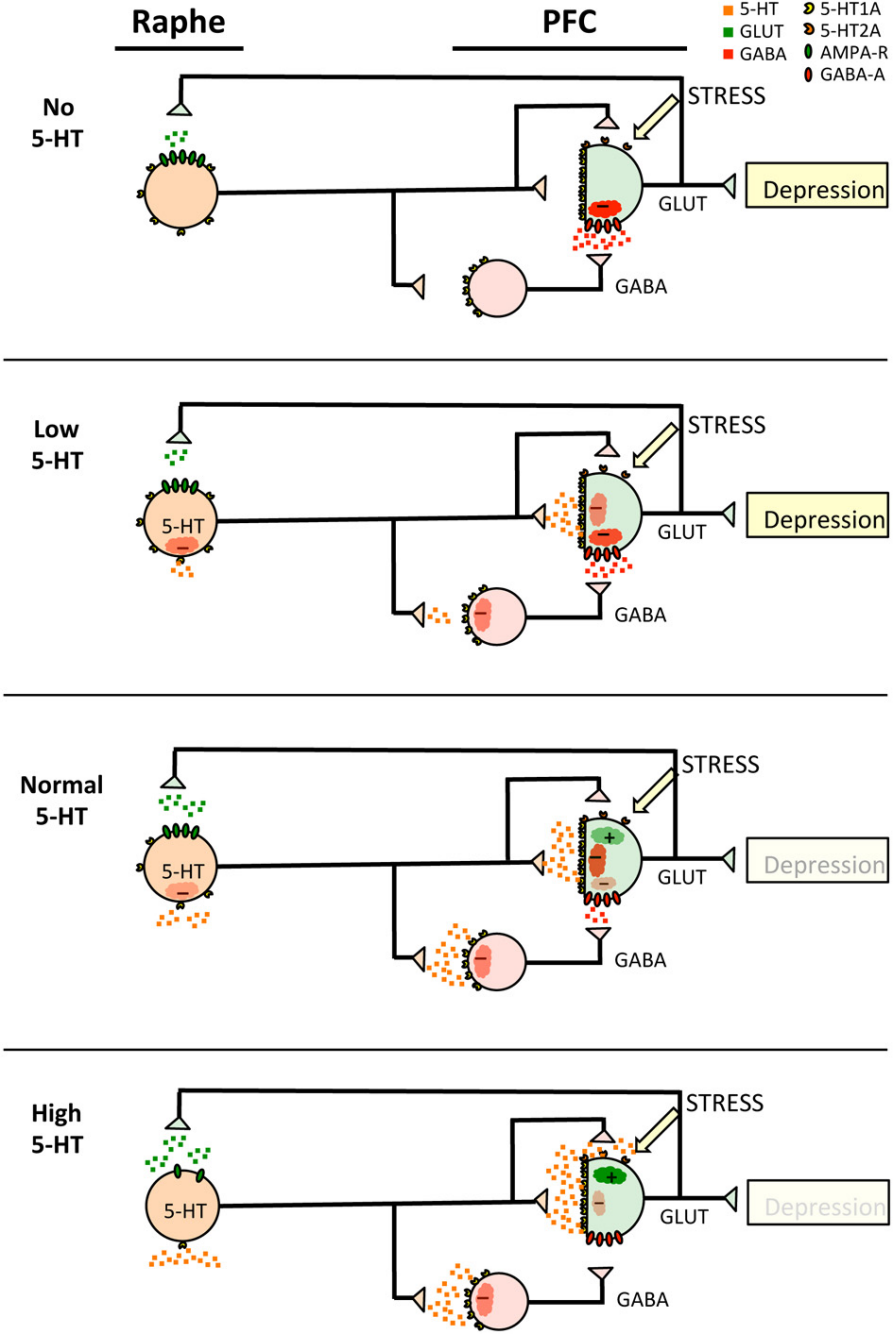
**Adult 5-HT-PFC circuitry in depression models**. Shown are the components of the Raphe 5-HT-PFC circuit in adult animals with no, low, normal or high levels of 5-HT neurotransmission as indicated. The model shows 5-HT neurons (orange) projecting to prefrontal cortex GABAergic interneurons (red) and glutamatergic pyramidal neurons (green) with transmitter release illustrated as small squares of the same colors. The presence of 5-HT1A (yellow), 5-HT2A (orange), AMPA-glutamate (green) and GABA-A receptors (red) is shown, as well as the response in the target neurons (clouds), stimulatory (+) or inhibitory (−). The effect of stress to stimulate pyramidal output is also indicated. It is postulated that 5-HT1A-mediated inhibition in adulthood becomes predominant in pyramidal neurons compared to interneurons, and that reduced activity of target pyramidal neurons elicits depression during adulthood.

Evidence from human imaging studies suggests that different sub-populations of PFC neurons with different targets mediate anxiety vs. depression behavior (Ressler and Mayberg, 2007; Krishnan and Nestler, 2008; Savitz et al., 2009). In addition, early post-natal development appears to be more important in the anxiety phenotype (see above) compared to the depression phenotype. Due to these differences, the serotonin circuitry model for depression involves similar components but is slightly different from the anxiety model (Figure 2). First, opposite to anxiety, activation of the pyramidal neuron is associated with reduced depression and increased resilience. Second, we propose that the 5-HT2A receptor is weakly active in the mature pyramidal neurons controlling depression (Beique et al., 2004). Based on this the major serotonergic control over the pyramidal neuron is through the pyramidal 5-HT1A receptor that reduces activity, but is almost saturated at normal 5-HT levels; at high 5-HT levels, 5-HT2A or other excitatory receptors (5-HT4, 5-HT7) are recruited to activate the pyramidal neurons and inactivate 5-HT1A signaling (Hedlund et al., 2005; Lucas et al., 2007). Finally, in this model the interneuronal 5-HT1A receptor is weakly active at normal 5-HT, hence is a target of SSRI to enhance inhibition of the interneuron thus further activating the pyramidal neuron. This model fits with genetic models involving loss or increase in 5-HT levels, and also with models of inhibition of adult 5-HT1A autoreceptor levels. For example, the TPH2^−/−^ and TPH2-R439H mutant that reduce or eliminate 5-HT show depression phenotypes (Jacobsen et al., 2012b; Mosienko et al., 2012; Sachs et al., 2013). Oppositely suppression of 5-HT1A autoreceptors in adult to increase 5-HT activity reduces depression behavior (Richardson-Jones et al., 2010; Bortolozzi et al., 2012; Ferres-Coy et al., 2013) (Table 1). Reduction of 5-HT1A autoreceptors in adulthood is associated with accelerated and increased release of 5-HT at target areas and enhanced SSRI-mediated behavioral improvement in depression and anxiety (Richardson-Jones et al., 2010; Bortolozzi et al., 2012; Ferres-Coy et al., 2013). This is consistent with the negative feedback role of 5-HT1A autoreceptors in regulating serotonergic activity, and the need to down-regulate their expression for SSRI to exert antidepressant activity (Albert and Francois, 2010).

However, the lack of effect on depression of lifetime suppression or over-expression of 5-HT1A autoreceptors (Richardson-Jones et al., 2011; Audero et al., 2013) is not accounted for in this model, suggesting that there may be early life adaptations to compensate for the over- or under-expression of 5-HT1A autoreceptors. Suppression of 5-HT1A heteroreceptors (using the CAMKIIα promoter to target pyramidal neurons) would be predicted to increase pyramidal neuron firing and display an anxiety phenotype rather than depression according to the model proposed. Autoradiography of the hetero-receptor suppressed mouse brain shows almost complete loss of cortical 5-HT1A binding (Richardson-Jones et al., 2011), suggesting that in this model CAMKIIα promoter is active in interneurons (as well as pyramidal cells), perhaps due to embryonic-initiated suppression. Reduced interneuron 5-HT1A receptors would enhance interneuron firing and inhibit pyramidal firing, leading to a depression phenotype similar to the low or no 5-HT condition according to the model (Figure 2). This result in Hetero-1A suppressed mice suggests that 5-HT2A excitation upon loss of 5-HT1A heteroreceptors is insufficient to drive the anxiety phenotype or prevent depression when the 5-HT1A autoreceptor is present.

Consistent with the above model for depression, there is evidence of a 50% reduction in major depression of calbindin-positive interneurons (which are 5-HT1A+ by in situ hybridization de Almeida and Mengod, 2008) and not of parvalbumin-positive interneurons (which lack 5-HT1A RNA) (Rajkowska et al., 2007). In this case, serotonergic activation would not reduce inhibitory interneuron feedback upon pyramidal neurons, leading instead to a further inhibition of pyramidal neurons, which could account for treatment resistant depression.

An aggressive phenotype is revealed in knockout models that produce a strong reduction of 5-HT (e.g., TPH2 knockout), but does not appear in 5-HT1A models (Table 1). Thus other 5-HT receptor subtypes may be involved, such as the 5-HT1B receptor (Saudou et al., 1994). In humans, aggressive behavior is more strongly associated with reduced 5-HT metabolites in cerebrospinal fluid than is depression, and in combination with depression, is a risk factor for suicide (Asberg, 1997). The presence of aggressive or impulsive behavior with depression seen in the “no 5-HT” (or very low 5-HT) condition, is consistent with findings of reduced 5-HT synthesis in aggressive subjects (Booij et al., 2010), and may increase likelihood of suicide attempt (Mann, 2013).

### Optogenetic studies of the serotonin-PFC circuitry in depression and anxiety

Optogenetic studies have provided important data for the formulation of serotonin-PFC circuitry models of anxiety and depression. For depression, specific optogenetic stimulation of rat PFC pyramidal neurons projecting to the raphe increased kicking frequency in the FST, indicating that these raphe-projecting pyramidal neurons drive the “antidepressed” response in this assay (Warden et al., 2012). These neurons are part of the long negative feedback system involving pyramidal 5-HT1A receptors that mediate inhibition of raphe activation by this projection (Figure 2). Oppositely, a different set of PFC neurons project to the lateral habenula to inhibit the kick response, inducing a “pro-depressive” state. Thus, different populations of PFC neurons may project to induce pro- or anti-depressant phenotypes, but it is not known whether the 5-HT1A receptor is expressed in the lateral habenula projecting neurons as it is in those that project to raphe. If not, modulation of 5-HT1A receptor activity in PFC would target the antidepressant response in a specific way.

Other studies suggest a similar circuitry to that proposed for PFC may exist in the hippocampal CA1 region (Varga et al., 2009). Optogenetic stimulation of raphe projections inhibits CA1 pyramidal neurons via 5-HT1A receptors on these neurons; in addition, raphe projections innervate interneurons activating them via 5-HT3 receptors, but also inhibiting them via 5-HT1A receptors. Further studies are needed to examine the specific contribution of 5-HT neurons to these actions. These studies suggest that the serotonin circuitry model that we propose for the PFC may be replicated in part in other brain regions such as the hippocampus that mediate serotonin-regulated behavioral and cognitive actions.

## Transcriptional modifiers of 5-HT1A receptor expression in anxiety and depression

Given the role of altered serotonin regulation in these genetic models of mouse behavior, what insights can be made into more effective treatment of human depression and anxiety by targeting different populations of the 5-HT1A receptor? One approach may be to “reset” the transcriptional regulation of the 5-HT1A receptor gene (Albert, 2012). The 5-HT1A receptor gene is regulated by a number of identified transcription factors, some of which show cell-specificity. For example, PET-1 is required for 5-HT1A receptor expression specifically in serotonin neurons due to its exclusive expression in these neurons, although it is not required in adult neurons (Liu et al., 2010). Other transcription factors that may dynamically regulate 5-HT1A receptors in the brain include Deaf1 and Freud-1/Freud-2 repressor proteins.

### The 5-HT1A C(-1019)G polymorphism and Deaf1

The human 5-HT1A C(-1019)G polymorphism (rs6295) is located upstream of the basal promoter in a palindromic DNA element that is recognized at the C-allele by transcription factors Deaf1 and Hes1/5 (Lemonde et al., 2003; Jacobsen et al., 2008). Hes1/5 expression in brain is restricted to early developmental neural progenitors and is switched off as they differentiate into mature neurons. Hes1 mediates stronger repression than Hes5, and gene knockout of Hes1 in mice results in premature embryonic over-expression of 5-HT1A receptors in the midbrain and hindbrain (Jacobsen et al., 2008). Deaf1 is expressed in the brain throughout development and in adulthood. Deaf1 represses 5-HT1A receptor expression in raphe cells (Lemonde et al., 2003), yet enhances its transcription in some non-serotonergic cells (Czesak et al., 2006). Importantly Deaf1 is strongly expressed in several regions that are enriched on 5-HT1A receptors, especially raphe nuclei, PFC and hippocampal CA1 pyramidal neurons (Lemonde et al., 2003; Szewczyk et al., 2009). In Deaf1^−/−^ mice, a 50% upregulation of 5-HT1A autoreceptors was observed, with a 30% reduction in 5-HT1A expression in PFC but no change in hippocampus, consistent with a neuron-specific regulation by Deaf1 (Czesak et al., 2012). Activation of Deaf1 could have beneficial effects: reducing 5-HT1A autoreceptor expression to enhance serotonergic activity while increasing 5-HT1A heteroreceptors in PFC. It is unclear however, whether 5-HT1A receptors on prefrontal pyramidal neurons, interneurons or both are affected by Deaf1. It is unclear whether Deaf1 is also expressed in interneurons. In septal SN-48 cells, which express 5-HT1A receptors and are GABAergic (Charest et al., 1993), Deaf1 enhances 5-HT1A receptor transcription (Czesak et al., 2006), suggesting that it may have enhancer activity on 5-HT1A receptors in interneurons but this remain to be addressed. Deaf1 is expressed in 5-HT1A+ pyramidal neurons of the human PFC, particularly lamina II/III (Szewczyk et al., 2009). In antidepressant-free female depressed subjects Deaf1 and 5-HT1A protein levels were decreased, consistent with reduced 5-HT1A-mediated inhibition of pyramidal neurons in depression that may be driven by reduced Deaf1 expression (Szewczyk et al., 2009). Interestingly, estrogen induces Deaf1 protein levels in cultured neuronal cells (Adeosun et al., 2012), which could underlie a more prominent role in female depression. Although activation of Deaf1 could be beneficial in treatment of anxiety and depression, Deaf1 may also have beneficial actions on the immune system including enhancing responses to peripheral tissue antigens and to viral antigens (Yip et al., 2009; Ordureau et al., 2013).

The 5-HT1A C(-1019)G polymorphism (rs6295) appears to alter 5-HT1A receptor expression in humans and is associated with major depression, suggesting a role for Deaf1 in human depression. In particular, this polymorphism has been associated with major depression (Lemonde et al., 2003; Kishi et al., 2013), bipolar depression (Kishi et al., 2013), completed suicide (Wasserman et al., 2006) (although not suicidal ideation or attempt) and anxiety disorders as well as reduced response to SSRI and antipsychotics that target 5-HT1A receptors (Le François et al., 2008; Newman-Tancredi and Albert, 2012). In one study, the strongest association was seen in patients with depression and comorbid anxiety (Molina et al., 2011). Adult stress, but not early life stress, in combination with the risk G-allele increases the association with increased stress reactivity, depression and suicide (Wasserman et al., 2006; Benedetti et al., 2011; Mekli et al., 2011; Lebe et al., 2013). However in a larger study of normal subjects no such associations were found (Chipman et al., 2010). PET imaging studies in non-medicated depressed subjects show that the GG genotype is associated increase 5-HT1A receptors in raphe, with little change in post-synaptic regions (Hesselgrave and Parsey, 2013). Others groups have reported decreases in post-synaptic 5-HT1A receptors in cortex, hippocampus and amygdala in depression or anxiety (Akimova et al., 2009). Treatment with antidepressants leads to a reduction in raphe 5-HT1A binding potential (Gray et al., 2013), and the GG-genotype ha been associated with reduced response or resistance to antidepressant treatment. Taken together these results implicate dys-regulation of 5-HT1A gene transcription in predisposition to depression and related disorders, and in reducing response to antidepressant treatment.

### Freud-1/Freud-2 repression of the 5-HT1A promoter

Freud-1 was identified as a strong repressor of 5-HT1A autoreceptors on raphe neurons, but also represses the receptor in non-serotonergic neurons (Ou et al., 2003). Freud-1 is strongly expressed in raphe, PFC and hippocampus and colocalized with 5-HT1A receptors. Freud-1 appears to be stress-sensitive, as chronic stress in rats reduced Freud-1 levels in PFC while increasing 5-HT1A receptor levels, perhaps representing a compensatory adaptation to the stress (Iyo et al., 2009). In humans, Freud-1 and 5-HT1A protein levels were reduced in PFC of depressed subjects particular younger subjects, suggesting an early role (Szewczyk et al., 2010). While Freud-1 protein was reduced, Freud-1 is inhibited by calcium-CaMK and could downregulate 5-HT1A expression in response to reduced calcium levels (Ou et al., 2003). Since CaMKII is primarily expressed in pyramidal neurons, calcium-mediated inactivation of Freud-1 may be more specific to these neurons compared to interneurons, leading to calcium-mediated up-regulation of 5-HT1A heteroreceptors specifically in pyramidal neurons.

Freud-2 is a homologue of Freud-1 and binds to a site adjacent to Freud-1 to repress 5-HT1A receptor expression (Hadjighassem et al., 2009, 2011). Unlike Freud-1, Freud-2 sparsely expressed in the raphe, but is strongly expressed in PFC where it is colocalized with 5-HT1A receptors and a trend for reduced Freud-2 in the PFC of depressed patients was observed (Hadjighassem et al., 2009). Down-regulation of Freud-2 may be beneficial in depression to increase 5-HT1A expression in pyramidal neurons and enhance their firing activity.

### Glucocorticoid-mediated repression of the 5-HT1A promoter

The 5-HT1A receptor gene is repressed by glucocorticoids, particular in the hippocampus which expresses both high and low affinity glucorticoid receptors (MR and GR, respectively). Together the MR/GR complex represses 5-HT1A promoter at a negative GRE, as well as indirectly by suppressing Sp1-induced promoter activity (Meijer et al., 2000; Ou et al., 2001). Studies in rodents have shown that post-synaptic 5-HT_1A_ receptors are impacted by stress or corticosteroids. 5-HT_1A_ receptors in the rodent hippocampus (Chalmers et al., 1994; Lopez et al., 1998; van Riel et al., 2003, 2004) and basolateral anterior, basolateral ventral and basomedial amygdaloid nuclei as well as the hypothalamus (Vicentic et al., 2006) are desensitized or down-regulated after chronic stress or corticosterone administration. In addition, socially stressed tree shrews have reduced 5-HT1A receptor levels in the posterior cingulate cortex, parietal cortex, hippocampus, and PFC. These stress-induced effects are prevented by adrenalectomy (Chalmers et al., 1994) and suggest a key role of stress in 5-HT1A receptor dys-regulation. Similarly, SSRI induced reduction in 5-HT1A autoreceptor binding is observed in chronic stress models but not naïve animals (Rainer et al., 2012). In human social anxiety, 5-HT1A receptors are reduced in amygdala and cingulate cortex and negatively correlated with plasma cortisol levels, consistent with glucocorticoid-induced down-regulation of the receptor in anxiety-associated brain regions (Lanzenberger et al., 2007, 2010).

Targeting elevated activity of the hypothalamo-pituitary axis or glucocorticoid signaling in anxiety and depression may have several beneficial effects via alteration in the serotonin system via up-regulation of 5-HT1A heteroreceptors, including a preferential effect to enhance hippocampal 5-HT1A receptors which are the most sensitive to glucocorticoids (Meijer and de Kloet, 1998). In models with chronic elevation of glucocorticoids, glucocorticoid inhibition would induce a more general up-regulation of 5-HT1A heteroreceptors throughout the brain, although the specificity for pyramidal vs. interneuron 5-HT1A receptors remain to be clarified.

## Conclusion: integrated model of 5-HT-prefrontal circuitry in anxiety and depression

Recent and increasingly sophisticated mouse genetic models have resulted in a more precise dissection of the roles of 5-HT activity, and 5-HT1A autoreceptors vs. heteroreceptors in anxiety- and depression-like behavior in rodents. These models have been examined for their anxiety and depression-like behavioral phenotypes and have shown that (see Table 1):

- Alteration in serotonin genes (5-HT1A, 5-HTT, TPH2) produces an anxiety and/or depression phenotype.
- Early post-natal modulation of the 5-HT system can produce lifelong changes in anxiety/stress reactivity.
- Both pre- and post-synaptic 5-HT1A receptors contribute to anxiety and depression phenotypes, often in opposite ways.
- High (5-HT1A autoreceptor or 5-HTT knockout) or low (TPH2 or Pet-1 knockout) 5-HT neurotransmission can result in anxiety phenotype; very low 5-HT results in anxiety and aggressive behavior.
- Downregulation of 5-HT1A autoreceptor accelerates and enhances SSRI action (Richardson-Jones et al., 2010; Bortolozzi et al., 2012).
- 5-HT1A receptors are required for neurogenic and anti-anxiety actions of chronic SSRI treatment (Santarelli et al., 2003).

In order to begin to integrate results obtained from these studies, we have proposed a model of 5-HT-PFC circuitry that takes into account the presence of 5-HT1A heteroreceptors on both interneurons and pyramidal neurons (Figures 1, 2). The model of anxiety considers that early post-natal circuitry is critical, while the depression model propose the role of a different population of PFC neurons during later development. The model postulates that behavioral phenotype shifts as serotonin activity increases:

- No or very low 5-HT: depression and aggression phenotype (high risk for suicidal behavior).
- Low 5-HT: anxiety (early post-natal) and depression (adolescent-adult).
- Normal 5-HT: no anxiety, aggression, and depression.
- High 5-HT: anxiety (early post-natal) phenotype.

Predictions of the model include the co-morbidity of anxiety and depression (at low 5-HT), alternation between depressed and anxious states (low to very low 5-HT), as well as the effectiveness of SSRI on both anxiety and depression (increasing 5-HT from very low or low to normal). Additionally, 5-HT1A-mediated inhibition of interneuronal firing is predicted to play a key role in activation of PFC pyramidal neurons that could either induce anxiety (during early post-natal period) or reduce depression in adult. The importance of this circuit in behavior remains unclear, in part because selective modulation of 5-HT1A receptors in PFC interneurons has not been achieved.

The specific role of interneuronal 5-HT1A receptors in regulating pyramidal neurons has been studied electrophysiologically, however their role in behavior has yet to be specifically addressed. An understanding of the role of both pyramidal and interneuronal 5-HT1A heteroreceptors in behavior and how their expression may change in human depression and anxiety may provide important insights into targeting distinct populations of 5-HT1A heteroreceptors by transcriptional modifiers. To date, interneuron-specific expression of CRE for conditional knockout is limited to interneuron subtype (calbindin, parvalbumin, or somatostatin), but not specific brain regions. Systemic administration of 5-HT1A agonists, such as DPAT, activates pyramidal firing preferential through the interneuronal pathway, but also inhibits 5-HT neurons via 5-HT1A autoreceptor activation (Llado-Pelfort et al., 2012). However, new generation 5-HT1A agonist show promise in targeting post-synaptic 5-HT1A receptors, and may prove selective to interneurons (Llado-Pelfort et al., 2010), and more effective as antidepressants than less selective 5-HT1A agonists such as buspirone (Rush et al., 2009). Alternately, pharmacological modification of transcription factors that show regions specific activity on 5-HT1A transcription, such as Deaf1 or MR, may provide new approaches to treat depression or augment antidepressant activity.

### Conflict of interest statement

The authors declare that the research was conducted in the absence of any commercial or financial relationships that could be construed as a potential conflict of interest.
